# Back to basics: locally produced vaccines offer a practical alternative to antibiotics for prevention of streptococcosis in farmed tilapia (*Oreochromis* spp.)

**DOI:** 10.3389/fvets.2026.1740757

**Published:** 2026-02-24

**Authors:** Nguyen Tien Vinh, Ha Thanh Dong, Jessica Kaye Turner, Saengchan Senapin, Warren Andrew Turner

**Affiliations:** 1 Aquaculture and Aquatic Resources Management, Faculty of Food, Agriculture and Natural Resources, Asian Institute of Technology, Pathum Thani, Thailand; 2 Nam Sai Farms Co., Ltd., Prachinburi, Thailand; 3 National Center for Genetic Engineering and Biotechnology (BIOTEC), National Science and Technology Development Agency (NSTDA), Pathum Thani, Thailand; 4 Fish Health Platform, Center of Excellence for Shrimp Molecular Biology and Biotechnology (Centex Shrimp), Faculty of Science, Mahidol University, Bangkok, Thailand

**Keywords:** alternatives to antibiotics, autogenous, cost–benefit analysis, *Streptococcus agalactiae*, tilapia, vaccine

## Abstract

While novel and advanced vaccine technologies offer significant potential for aquaculture, their adoption is often limited by high costs, particularly in low-value species like tilapia, underscoring the value of simpler approaches. *Streptococcus agalactiae* is a major pathogen in tilapia farming, causing significant economic losses. While vaccination offers protection, commercial vaccines often show inconsistent efficacy due to serotype variation, regional strain shifts, and limited availability in Southeast Asia. The objective of this study was to establish and evaluate a simple, locally adaptable, and back-to-basic vaccination strategy using two bivalent formulations, heat-killed vaccine (HKV) and formalin-killed vaccine (FKV), against *Streptococcus agalactiae* serotypes Ia and III. Tilapia received a primary intraperitoneal injection followed by a booster dose after 4 weeks. In the lab, vaccinated fish exhibited sustained IgM antibody responses against both serotypes for 84 days, with peak levels on days 35–42 after the booster. Challenge trials with lethal doses demonstrated high relative percent survival (RPS), reaching 97.4–100% for serotype Ia and 86.8–97.4% for serotype III, showing comparable protection between HKV and FKV. Given its simple preparation, scalability, safety, and rapid production without the use of toxic chemicals while retaining bacterial extracellular products, the HKV vaccine was further evaluated on a commercial farm. The HKV elicited a significant serum IgM antibody response in vaccinated tilapia, which remained elevated for up to 126 days and conferred protection during a natural disease outbreak. At the end of our six-month trial, survival rate in the vaccinated treatment was on average 94.5%, significantly higher than the control group at 66.8%. Moreover, the vaccine application significantly lowered feed conversion ratio, increased total biomass, and enhanced revenue by approximately 45%, resulting in higher profit. These findings demonstrate that the basic bivalent HKV is an effective, practical, and locally feasible alternative to commercial vaccines for controlling streptococcosis in tilapia, offering a promising strategy for widespread adoption in resource-limited aquaculture settings.

## Highlights

- Bivalent *S. agalactiae* HKV and FKV triggered comparable antibody responses in tilapia.
- Serotype Ia induced higher antibody levels than serotype III.
- Both vaccines demonstrated high efficacy against both serotypes under laboratory conditions, with RPS ranging from 86.8 to 100%.
- The HKV conferred high effectiveness under farm conditions with a RPS of 83.4%.
- On-farm implementation of HKV prevented streptococcosis outbreaks, resulting in significantly lower FCR, increased total biomass and higher profitability at harvest.

## 1 Introduction

Tilapia (*Oreochromis* spp.) are a major family of species in aquaculture, accounting for about 10.7% of global freshwater fish production in 2023 (1). Their rapid growth and high adaptability to different water conditions and farming systems make them ideal for aquaculture, particularly in low to middle-income countries (2, 3). Farmed in over 135 countries and territories, tilapias play a significant role in sustainable aquaculture and global food security, with production of approximately 6.8 million metric tons in 2023 (1, 4). However, intensive tilapia farming has been increasingly challenged by disease outbreaks, hindering further expansion of the industry (5–11).

Streptococcosis, causing septicemia and meningoencephalitis, is one of the most serious infections in tilapia, particularly during hot weather. *Streptococcus agalactiae* (also called GBS – Group B Streptococcus) is the leading cause of the disease in tilapia that frequently drives high mortality and severe economic losses in tilapia aquaculture worldwide (12, 13). Based on the capsular polysaccharide structure, at least 10 serotypes (Ia, Ib, III to IX) of *S. agalactiae* have been classified. Among them, serotypes Ia, Ib, III (14), and more recently serotype IV has been reported to cause disease outbreaks in tilapia (15). Serotypes Ia and III are most associated with streptococcosis in Thailand (16–18). Tilapia infected with *S. agalactiae* usually exhibit swirl swimming, exophthalmia, loss of appetite, external petechial hemorrhages, swollen internal organs and ascites (9, 10, 19). Heavy infections of *S. agalactiae* may cause up to 50–70% mortality (9, 10). In tilapia farming across Asia, it was estimated that *S. agalactiae* alone accounted for an annual loss of approximately $500,000 (20).

Vaccination represents a key strategy for mitigating the burden of infectious diseases and reducing reliance on antibiotics, thereby helping to prevent the emergence and spread of antimicrobial resistance (21–24). A vaccine contains a biological preparation that elicits specific immunity of the fish, protecting them upon exposure to the same pathogen(s). For tilapia, numerous vaccine formulations against *S. agalactiae* have been developed and tested in the laboratory, including live attenuated vaccines (25–27), inactivated vaccines (28–32), subunit vaccines (33, 34), and DNA vaccines (35–37). In the market, streptococcosis vaccines are primarily formalin-killed vaccines, consisting of single- or multiple- serotypes that are administered by injection. Available vaccines include ALPHA JECT® micro 1 Tila,^1^ AQUAVAC® Strep Sa,^2^ and Virbac S. A^3^ (38). However, vaccine coverage in tilapia farms is low (<5%), due to the lack of accessibility, cost, and the uncertainty regarding the effectiveness (39). Thus, there is still a strong demand for locally applicable vaccines for low-value species like tilapia.

An autogenous vaccine is a custom vaccine made from pathogens isolated from a specific outbreak or farm to protect that same population, particularly when commercial vaccines are unavailable or poorly matched to circulating strains (40). Barnes et al. (41) noted that autogenous vaccines, locally produced as part of a targeted veterinary health program, can offer a cost-effective way to reduce disease and antibiotic use. The presence of multiple serotypes of *S. agalactiae* can reduce vaccine efficacy, emphasizing the importance of multivalent formulations for broader protection. Additionally, vaccines may be less effective against non-homologous antigens (42, 43). Therefore, vaccines tailored to homologous, locally prevalent serotypes and strains may be needed to improve protective efficacy.

Previous research has reported that a formalin-killed bivalent *S. agalactiae* vaccine (serotypes Ia and III) was highly effective in Nile tilapia farms (44). Although formalin is widely used to inactivate bacteria for vaccine production, this approach poses potential risks to users and the environment. Moreover, large-scale production typically requires specialized infrastructure to remove formalin residues, while the process can result in the loss of secreted soluble bacterial proteins. In some cases, formalin inactivation may also reduce the antigenicity of the vaccine (45–47). On the other hand, an alternative heat-killed streptococcosis vaccine (HKV) has not been well studied, likely due to concerns that heat-induced antigen denaturation could reduce immunogenicity. Despite this, the approach offers simple preparation, scalability, safety, and rapid production without toxic chemicals, while potentially preserving bacterial extracellular products. These advantages could make HKV a viable and cost-effective vaccine option for streptococcosis. However, evidence comparing the protective efficacy of heat-killed streptococcosis vaccines with formalin-killed vaccines (FKV) in controlled environments remains limited. Moreover, the field performance of HKV - including protection and cost-effectiveness - has yet to be documented.

In this research, we evaluated a locally produced autogenous bivalent heat-killed vaccine (HKV) targeting *S. agalactiae* serotypes Ia and III, administered via injection for farm application. The research comprised of two stages – a lab experiment and a small-scale field trial. The lab experiment provided the evidence of the vaccine’ efficacy via specific antibody elicitation and the survival of tilapia following bacterial challenge. The farm trial was strategically conducted during the peak incidence of *S. agalactiae* infections, which corresponded to the hot season in Thailand from April to June, thereby enabling an evaluation of vaccine effectiveness in reducing mortality under high-risk conditions.

## 2 Materials and methods

### 2.1 Fish stock and husbandry practice

The use of fish in this study complies with the ethical guidelines approved by the Animal Ethics Committee of Mahidol University (No. MUSC67-053-758). Four hundred and fifty healthy Nile tilapia juveniles (26.3 ± 9.1 g) from the Aquaculture and Aquatic Resources Management (AARM) hatchery were used for the lab experiment. Before the vaccine immunization, the fish were allowed to acclimatize for 7 days in a wet lab. During the vaccine trial, the fish were kept in 1000 L circular polyethylene tanks, at a stocking density of 150 fish per tank. The fish were fed twice per day at 3% body weight (Lee Feed Mill Public Company Limited - Win 32, 32% crude protein). Each tank was equipped with a physical filter and air stone aeration. Seventy percent of the water was exchanged every two days. The water condition was maintained as follows: dissolve oxygen (DO) 4–6 ppm, temperature 28–30 °C, pH 6.5–7.5, total ammonia nitrogen (TAN) < 0.5 ppm, nitrite (NO_2_) < 0.1 ppm, nitrate < 0.1 ppm, salinity 0 ppt.

For the field vaccine trial, hybrid red tilapia (*Oreochromis* sp.) was chosen over Nile tilapia (*O. niloticus*) due to their relevance in current aquaculture practices. Both groups are known to be susceptible to *S. agalactiae*, and the popularity of hybrid red tilapia in Thailand makes this choice more applicable to real-world farming conditions.

In total, 2,400 hybrid red tilapia (approximately 40 g each) were housed in eight cages (dimensions: 4 m x 3 m x 1.2 m deep), which were placed in a 2.3-hectare freshwater reservoir. Feeding practices were consistent with the standard operations of the farm to reflect real-world aquaculture conditions. The fish were fed till satiation three times a day using 4 mm floating tilapia pellets at 8:00 a.m., 1:00 p.m., and 4:30 p.m. Aeration was provided during the night (from 10:00 p.m. to 8:00 a.m.) and during the day (from 11:30 a.m. to 1:30 p.m.) using blowers and disk diffusers in each cage to ensure adequate dissolved oxygen levels. Water quality inside cages at a depth of 50 cm was measured twice a week at 3:00 p.m. at two different points, assessing pH (unit), DO (ppm), temperature (°C), TAN (ppm), and NO₂ (ppm). During the field trial, no antibiotics were used.

### 2.2 Vaccine preparation

Among 134 *S. agalactiae* isolates collected from tilapia cultured across multiple provinces in Thailand between 2018 and 2024, molecular serotyping identified serotypes Ia and III in equal proportions (unpublished laboratory data). Two representative strains, 3,868 (serotype Ia) and F2S (serotype III), isolated from disease outbreaks in Prachinburi Province, Thailand, were selected for vaccine preparation and challenge experiments. For preservation, the bacterial cultures were stored at −60 °C in 20% (v/v) glycerol until use.

The vaccines were prepared as previously described (48) with minor modifications. Firstly, a single colony of each bacterial strain was initiated in 20 mL tryptic soy broth (TSB) in a 50 mL conical centrifuge tube and incubated at 32 °C for 8 h., with continuous shaking at 150 rpm. They were then upscaled by transferring 1 mL of bacterial culture to a new sterile 500 mL flask containing 200 mL TSB. After culturing at 32 °C for 15 h., the final bacterial concentrations were measured by triplicate plate count on tryptic soy agar (TSA), along with optical density measurement at 600 nm (OD600).

Two types of inactivated vaccines were prepared – heat-killed vaccine (HKV) and formalin-killed vaccine (FKV). For HKV, the bacterial culture was inactivated in a water bath at 60 °C for 1 h. The FKV, on the other hand, was inactivated by adding 2% (v/v) of 37% stock formalin and incubated at room temperature for 2 h. The flask was then further incubated at 4 °C overnight (approximately 8 h.). The inactivation of both vaccines was validated by plating on TSA (triplicate) and incubated at 30 °C for 48 h. No colonies present on all three TSA plates confirm successful inactivation. For FKV, inactivated bacterial cells were collected by centrifugation at 4,000 rpm for 10 min. That was followed by washing 3 times with 1X PBS, pH 7.4. Finally, the bacterial cells were resuspended in 1X PBS by the same volume of the original culture. In HKV preparation, the whole bacterin containing bacterial cells and their extracellular materials were retained (no separation and washing were required).

The bivalent vaccines were prepared by mixing two inactivated *S. agalactiae* serotypes to reach a total concentration at ~3.05 × 10^9^ cells/mL. The bivalent HKV and FKV were stored at 4 °C until use for vaccination.

### 2.3 Lab scale trial of bivalent vaccines in nile tilapia

**Figure 1:**
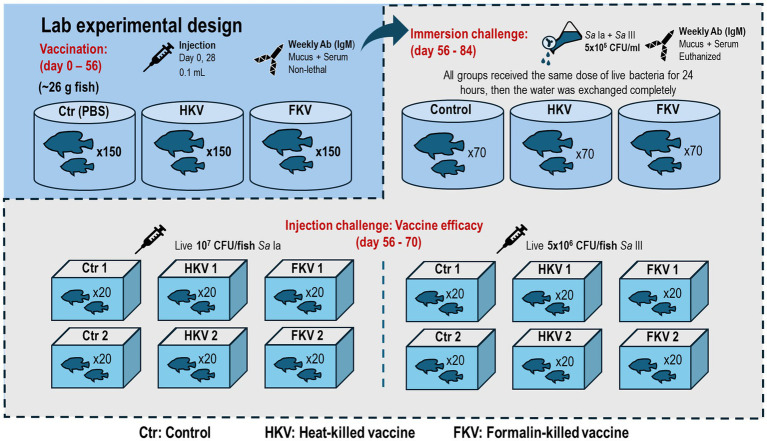
Experimental design in the lab experiment. The fish were injected with a primary dose and a booster dose on day 28. On day 56, a subset of fish was challenged intraperitoneally with separate serotype to determine vaccine efficacy. The other subset was challenged by immersion with mixed serotypes to further assess antibody response (IgM) via epithelial exposure.

The lab-scale experiment aimed to assess the efficacy of HKV and FKV to determine which vaccine is suitable for farm application. Prior to the experiment, five representative tilapia were screened for *S. agalactiae* by culture on tryptic soy agar (TSA) and by specific qPCR of head kidney tissue (80). Healthy Nile tilapia juveniles were divided into three groups, each consisting of 150 fish housed in a 1,000 L tank (Figure 1). The vaccine was administered by intraperitoneal injection with a primary dose of 0.1 mL on day 0, and a booster dose of 0.1 mL after 28 days. Before each injection (including vaccine administration and injection challenge), the fish were anesthetized using clove oil at 100 ppm.

The groups were assigned as follows: control (receiving no vaccine), HKV (fish received bivalent heat-killed vaccine), and FKV (fish received bivalent formalin-killed vaccine). The fish were kept for 56 days post vaccination (dpv) to monitor weekly antibody response (*n* = 6 per group). On day 56, 80 fish per group were transferred to smaller tanks for an injection challenge and monitored for 14 days. The remaining 70 fish in each group, on the other hand, were retained in their original tank for an immersion exposure with live mixed serotypes of *S. agalactiae* (Figure 1).

The injection challenge was aimed to assess vaccine efficacy. Challenge doses to estimate the median lethal dose (LD50) were determined by intraperitoneal injection of live bacteria from each serotype at 10^6^, 5 × 10^6^, and 10^7^ CFU/fish (n = 10 per dose). The fish were then kept for 14 days. In each group, the experimental fish were divided into two subgroups, each subgroup consisting of two replicates (*n* = 20). One subgroup received a lethal dose of *S. agalactiae* Ia at 10^7^ CFU/fish, while it was 5 × 10^6^ CFU/fish for serotype III. The fish were monitored three times per day (at 8 a.m., 2 p.m. and 6 p.m.) and recorded mortality over a 14-day period. Representative moribund fish (at least 3 per subgroup) were collected to confirm the presence of *S. agalactiae* in the liver and head kidney by re-isolation on TSA plate.

The immersion exposure was designed to compare the antibody response between the vaccinated and non-vaccinated fish when exposed to a sublethal concentration of *S. agalactiae* in water. The water was reduced to 200 L before adding the mixture of *S. agalactiae* serotype Ia and III. The final concentration of bacteria was adjusted to 5 × 10^5^ CFU/mL per serotype to mimic the extreme conditions of high pathogen load in water during the disease season. This selected concentration was substantially higher than *Streptococcus* spp. levels reported in aquaculture water, which ranged from approximately 5 × 10^2^–9 × 10^3^ CFU/mL (49), but similar to an immersion challenge dose (10^6^ CFU/mL) reported to induce infection while causing negligible mortality in tilapia (50). The fish were exposed to the high bacterial load for 24 h., after which the water was drained completely and replaced with new fresh water. The fish then were reared in the same condition as described in section 2.1, with antibody surveillance (*n* = 6 per group) on 59, 63, 70, 77 and 84 dpv.

All water potentially contaminated with *S. agalactiae* was disinfected with chlorine (20 ppm) prior to discharge, in accordance with institutional biosecurity procedures.

### 2.4 Field trial of the bivalent vaccine in red tilapia

**Figure 2:**
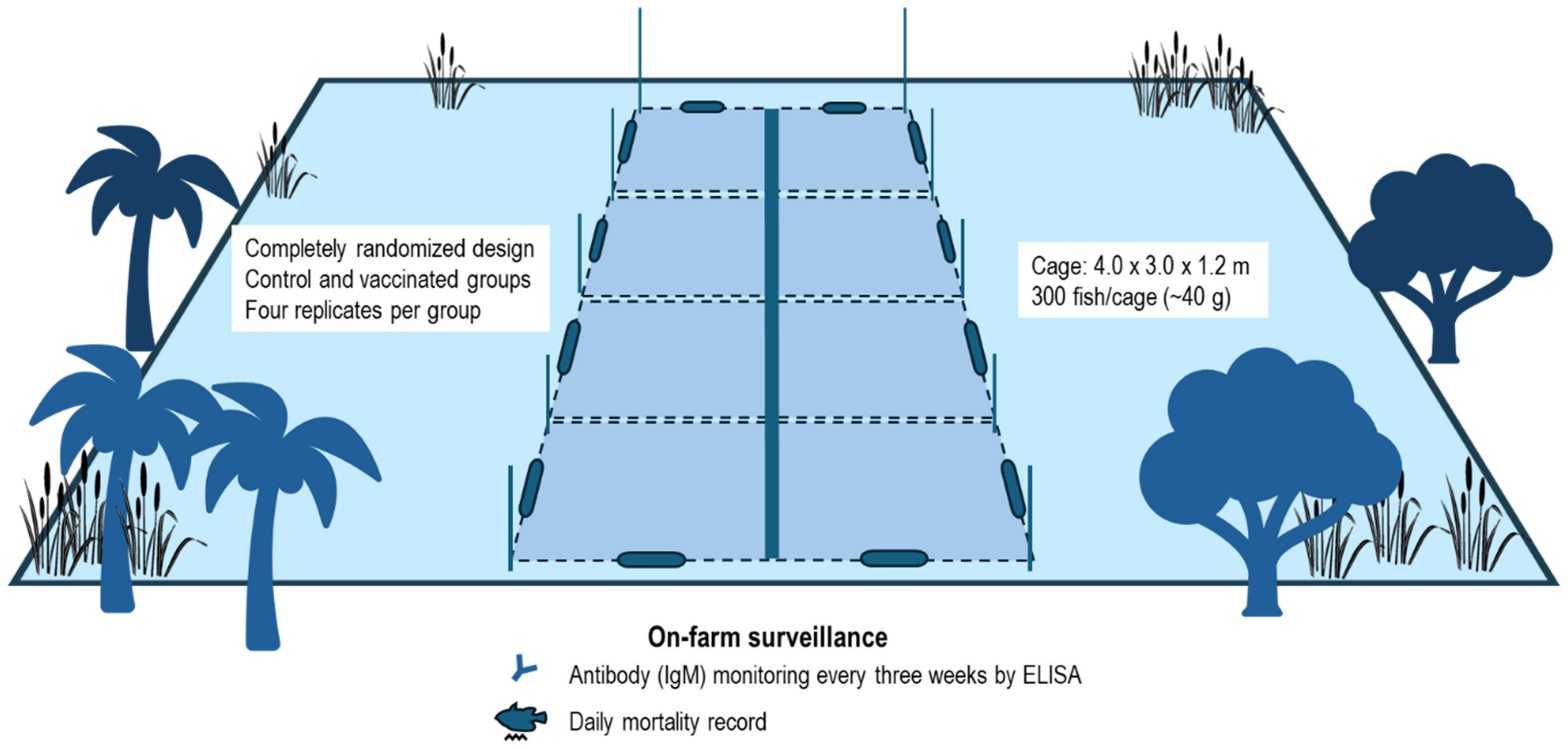
Farm vaccine trial design in floating net cages. Each fish group – control and vaccinated, were separated into four replicates containing 300 fish. Daily mortality monitoring was conducted with monthly antibody assessment and growth performance.

Based on their comparable efficacy in reducing mortality in the injection challenge and the relative ease of preparation, only the HKV was selected for the field trial. The field trial was performed in a six-month time frame at a commercial farm during the peak occurrence of streptococcosis. An overview of the experimental design is shown in Figure 2. The fish were administered two injections with a primary dose on day 0 and a booster dose on day 29. Because fish in the farm trial were larger, and to account for potential injection error by farm workers (e.g., vaccine leakage), the injection volume was increased to 0.2 mL per fish. The fish were divided into two groups – control (receiving no injection) and vaccinated (receiving HKV) to assess the vaccine effectiveness. Each group contained four replicates (*n* = 300) that were randomly assigned in cages.

During the trial, antibody surveillance was performed every 3 weeks (*n* = 3 per replicate). Fish mortality was observed and recorded every day. In addition, all fish in each cage were batch-weighed and counted every 4 weeks to determine growth expressed as average biomass per cage (kg), average fish weight (g), average daily gain (g/day), and feed conversion ratio (FCR). Average daily gain was calculated as [total biomass of the current sampling point – total biomass of the previous sampling point] / Number of days between sampling points. Cumulative FCR was determined as [cumulative feed use (kg)] / [total biomass (kg) at the current sampling point].

To evaluate the impact of the vaccination program on profit, a cost–benefit analysis (CBA) was performed using production costs and revenue at harvest. Production costs accounted for the main variable expenses, including vaccine, injection, seed, and feed costs. To streamline calculations, costs that were identical across treatments, such as cage depreciation electricity and feeding labor, were excluded from the calculation. Therefore, in this research, all cost–benefit analyses are based on incremental values, including costs, revenue, profit, profit-cost ratio (PCR), and profit margin.

Vaccine price was determined at 1,524 THB/L (including profit margin at 50%), with each 0.2 mL dose costing 0.3 THB (Appendix Table A1). The overall cost of implementing the vaccination program was estimated as 3,784 THB (Appendix Table A2). Crop revenue was derived from the net income per kilogram of fish (regardless of fish size), calculated as the selling price minus harvest and transport costs, with an average value of 70 THB/kg.

### 2.5 Sample collection

Fish body mucus and serum were non-lethally collected for antibody surveillance. Both mucus and serum were collected from each group per sampling point in the lab experiment, while only blood serum was taken from fish in the field trial. Before collecting samples, the fish were anesthetized with clove oil (100 ppm) to reduce stress during the procedure. To collect mucus, the fish was put in a plastic bag containing 1 mL 1X PBS and rubbed gently for 30 s to slough off the mucus. The mucus was then drawn into a 1.5 mL microtube and centrifuged at 10,000 rpm for 10 min to remove debris and insoluble materials. The supernatant was then transferred to a new tube and stored at -20 °C for further analysis. For fish serum, fish blood was first drawn from its caudal peduncle (around 100 μL per fish). The blood was allowed to settle at room temperature for 2 h., then centrifuged at 10,000 rpm for 10 min. The fish serum accumulated in the upper layer was then transferred to a new tube and stored at -20 °C until downstream analysis. All fish were released back to their respective tank or cage after sample collection. Fish that were exposed to *S. agalactiae* in the lab experiment, however, were humanely euthanized by an overdose of clove oil (200 ppm) and autoclaved to comply with the biosecurity protocol.

### 2.6 Fish antibody surveillance by enzyme-linked immunosorbent analysis

ELISA was employed to monitor the temporal changes of anti-*S. agalactiae* antibody in fish as described in a previous study (30). Briefly, a microplate was first coated with 100 μL of either antigen (prepared from FKV, see section 2) at 10^8^ cells/mL that was diluted by 10 times in carbonate–bicarbonate coating buffer. The plate was then incubated at 4 °C overnight (around 8 h.). The following day, the plate was washed (each three times) with 100 μL of 1X PBS + 0.05% Tween-20 (pH 7.4). This was followed by the addition of 100 μL of diluted fish mucus (8 times) or serum (512 times). The procedure continued with alternative washing and adding of reagents (100 μL each) as follows: primary antibody - mouse anti-tilapia IgM antibody (1: 200, Marine Leader Ltd., Co., Thailand), secondary antibody - goat anti-mouse IgG HRP conjugate (1:3000), TMB substrate, and finally stopping agent 2 M H_2_SO_4_. The signals were read at the wavelength of 450 nm (OD450) in a microplate reader (AC3000, Azure Biosystems, United States).

### 2.7 Statistical analysis

All analyses were performed in R with RStudio. The difference of antibody response among fish groups on the same sampling point was performed with Kruskal-Wallis’s test, followed by Dunn’s multiple range test. The difference between comparisons was considered statistically significant when the adjusted *p-value* (Bonferroni method) was less than *0.05*. In addition, the differences in antibody level within one group in the lab trial were compared between two sampling points 28–35 (after booster), and 56–59 (after immersion exposure) using the same method. Based on the antibody assay of the fish before immunization (*n* = 18), a cut-off threshold that indicates positive specific antibody readings was calculated at the upper 95% interval (51).

Vaccine efficacies against individual serotype in the lab and vaccine effectiveness in the farm trial were calculated as relative percent survival: RPS = [1 – (% mortality in vaccinated/ % mortality in respective control)] × 100. In the lab, the differences in survival rates among fish groups were compared using a log-rank test at a significance level of *p < 0.05*, as demonstrated by Kaplan–Meier curves. In the field, endpoint survival rates between control and vaccinated groups were compared using a chi-square test, with the significance level determined at *p < 0.05.* Student’s t-test was employed to compare production and growth data between control and vaccinated groups in the field trial at a significance level *p < 0.05.* Water quality data is presented in ranges of each parameter with average values throughout the crop period. Meanwhile, CBA was calculated using the total values of each group.

## 3 Results

### 3.1 Fish IgM antibody response in the lab experiment

#### 3.1.1 Serum antibody

**Figure 3:**
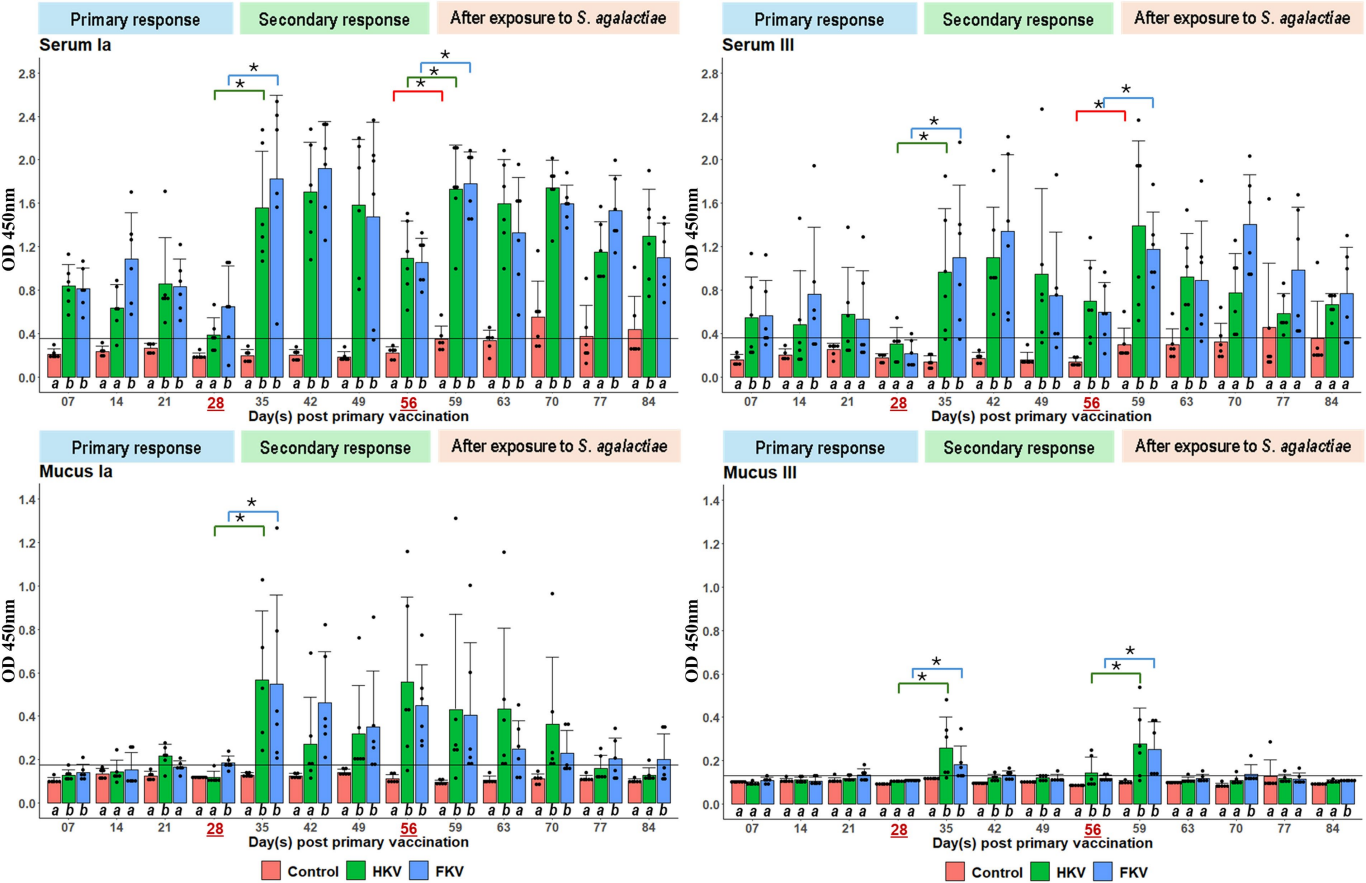
IgM antibody assay by ELISA (diluted by 512 times for serum and 8 times for mucus) of fish in the lab trial. The booster and the immersion exposure to a sublethal dose of live *S. agalactiae* were performed on day 28 and 56, respectively. Each dot represent one biological replicate (*n* = 6). Different alphabets below each bar on the same time point indicate statistically significant difference (*p < 0.05*) among treatment groups. Connectors with asterisk marks indicate significant difference (*p < 0.05*) in antibody response between two time points of the same treatment group (matched with respective colors). Horizontal lines represent cut-off values calculated at 95% upper interval of all OD measuring of fish before the trial (*n* = 18).

Figure 3 demonstrates the serum-specific antibody response (IgM) of the fish to *S. agalactiae* serotype Ia and III following primary immunization, booster administration, and subsequent immersion exposure. A similar antibody response pattern was observed in both HKV and FKV groups. After the first 7 days of vaccine injection, the significant antibody response was observed in both serotypes and vaccine types. Before the booster dose on day 28, OD measuring was only significantly different (*p < 0.05*) in FKV against serotype Ia compared with the corresponding control. In contrast, antibody levels against serotype III in both vaccinated groups did not show a statistically significant difference from those in the control group at the sampling points on days 21 and 28; nevertheless, the values remained above the established cut-off threshold.

After receiving the booster dose, a significant rise of antibody in both HKV and FKV groups on day 35 (7 days after the booster) was found. The levels of OD measuring in all vaccinated groups were maintained significantly higher than the control group till day 56. The highest average OD values were observed during this period as well, at 1.92 ± 0.43, on day 42 for serotype Ia, and 1.34 ± 0.71 for serotype III of the same day. Afterward, the values gradually decreased (but still significantly higher than control) on day 56.

Following exposure to a sublethal dose of live *S. agalactiae*, previously vaccinated fish exhibited a rapid rebound in antibody levels within just 3 days. In the vaccinated groups, OD measurements for both serotypes Ia and III remained above the established cut-off values and were significantly higher than those observed in the control group throughout the experimental period, up to 84 dpv, except for the final 2 weeks, during which the difference in antibody levels against serotype III was not statistically significant. In contrast, the control group displayed a gradual increase in average anti-*S. agalactiae* antibody levels for both serotypes following bacterial exposure (Figure 3).

#### 3.1.2 Mucus antibody

Specific antibody response in the mucus of the fish post vaccination and immersion challenge is shown in Figure 3. There were relatively low and inconsistent antibody response among vaccinated fish against serotype Ia after primary immunization. In contrast, no antibody response against serotype III was detected. After the booster dose, both HKV and FHV groups resulted in significant rise of mucosal IgM levels against both serotypes (*p < 0.05*), prolonging for the next 3 weeks (35, 42, 49 dpv). However, the peak average OD measuring against serotype Ia was two-fold higher compared to that of serotype III, e.g., 0.57 ± 0.32 versus 0.26 ± 0.14. The immersion exposure stimulated a noticeable increase of mucosal IgM against both serotypes. However, antibody against serotype III dropped rapidly after 1 week (63 dpv). In contrast, significant IgM levels against serotype Ia persisted until 84 dpv in FKV and 70 dpv for HKV.

### 3.2 Vaccine efficacies in the lab experiment

In the immersion exposure with a sublethal dose of mixed serotypes, only 3 out of 70 fish in the control fish died 1 week following the exposure (data not shown). In all groups (excluding the mentioned dead fish) did not exhibit obvious clinical signs of *S. agalactiae* infection till the end of the challenge.

**Figure 4:**
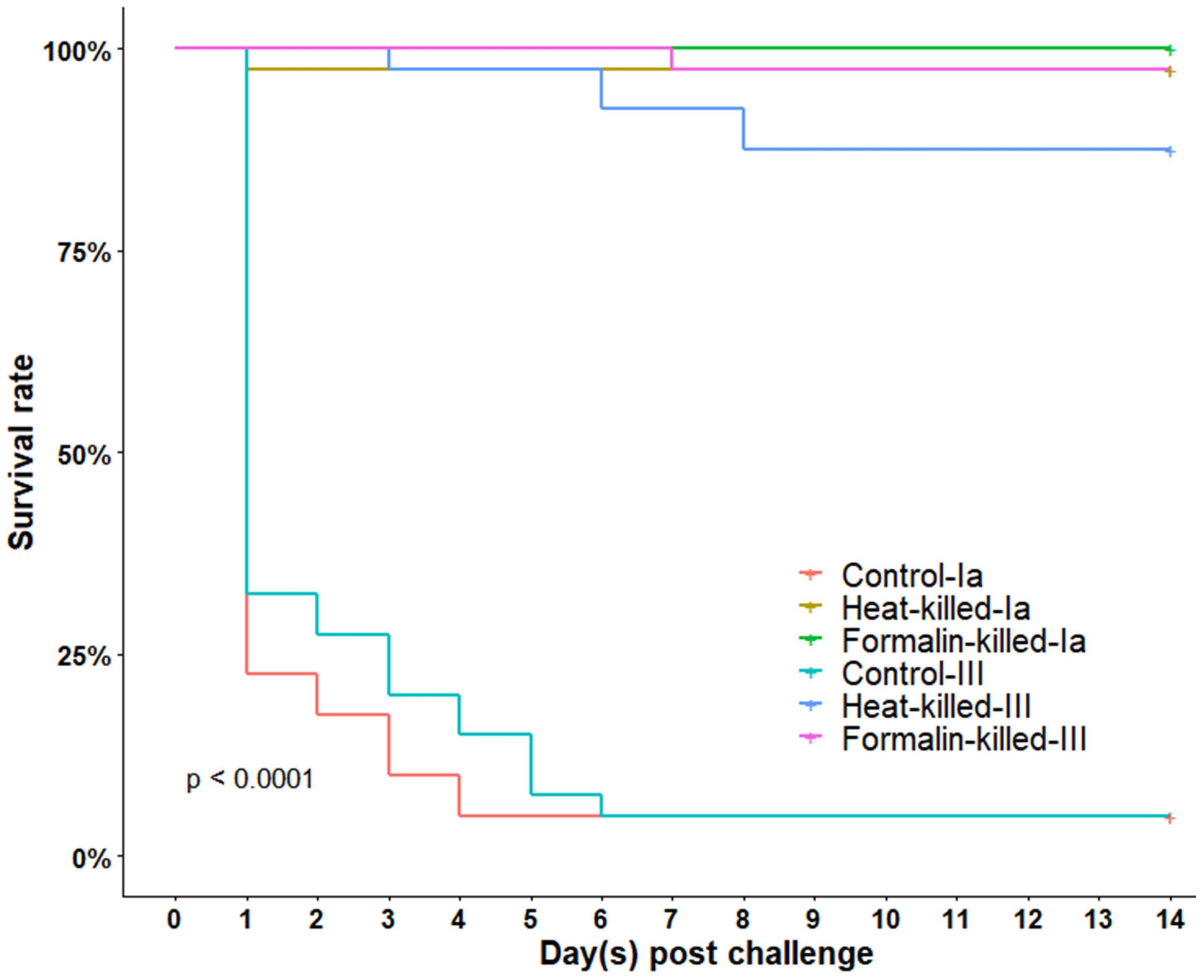
Kaplan–Meier graph showing survival rate of fish in the 14-day injection challenge in the laboratory experiment.

A fraction of fish was subjected to an injection challenge after 56 days of vaccination. After the injection with a lethal dose of *S. agalactiae*, mortalities occurred rapidly in the control groups in the first 24 h., reaching approximately 75% (Figure 4). The remaining survivors became moribund and died sporadically until day 6 post challenge. Moribund fish exhibited swirl swimming and edema after 3–4 days of injection. From these fish, white color pinpoint colonies were able to be isolated from the liver and head kidney. Interestingly, among surviving fish, clinical signs (pop-eyes and swollen belly) only manifested in the challenged non-vaccinated control groups. Vaccinated fish almost appeared apparently normal following the injection.

In the end, the survival rates for control fish injected with serotype Ia and III were both 5 ± 5% (Table 1). All vaccinated groups, on the contrary, displayed significantly high survival rates (*p < 0.05*), from 87.5 ± 2.5 to 97.5 ± 2.5 for serotype III, and 97.5 ± 2.5 to 100 ± 0% for serotype Ia. Hence, the corresponding RPS achieved 86.8–97.4% in fish groups challenged with serotype III, and 97.4–100% in fish groups challenged with serotype Ia. By the log-rank test, there was no significant difference (*p > 0.05*) in survival between HVK and FKV.

**Table 1:** Survival rates (mean ± SD) and RPS among fish groups after injection challenge in the lab.

Group	Survival rate (%)		RPS (%)	
	Ia	III	Ia	III
HKV	97.5 ± 2.5^b^	87.5 ± 2.5^b^	97.4	86.8
FKV	100 ± 0.0^b^	97.5 ± 2.5^b^	100	97.4
Control	5.0 ± 5.0^a^	5.0 ± 5.0^a^	–	–

Different alphabetical letters of the same column indicate statistically significant differences between groups (*p* < 0.05).

### 3.3 Fish IgM antibody response in the field trial

**Figure 5:**
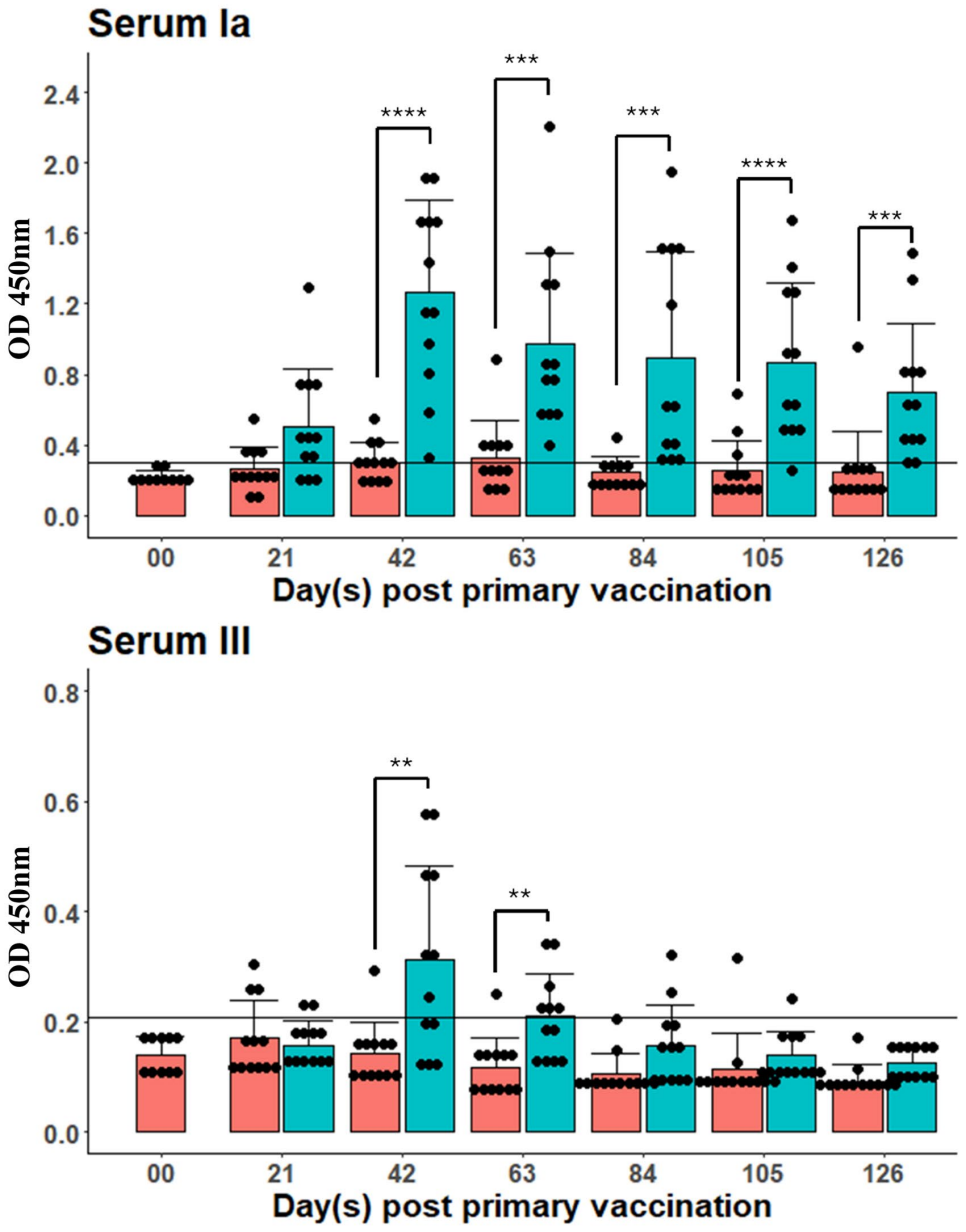
IgM antibody assay by ELISA of fish serum (diluted by 512 times) in the field trial. The booster dose was administered on day 29. Each dot represent one biological replicate (*n* = 12). Connectors with asterisk marks represent statistically significant difference between control and vaccinated group. Horizontal lines represent cut-off values calculated at 95% upper interval of all OD measuring of fish before the trial (*n* = 12). Significant levels: *p* < *0.05, **0.01, ***0.001, ****0.0001.

In the field trial (Figure 5), the cut-off threshold at significant level *p = 0.05* was established according to the OD measuring of serum IgM of all fish of the control group on day 0. Against serotype Ia, the vaccinated group displayed significant antibody response (*p < 0.05*) in all sampling points from day 42 till the end of the trial on day 121. In contrast, the IgM levels against serotype III only appeared on 42 and 63 dpv. The highest IgM levels recorded against both serotypes were on 42 dpv (e.g., 2 weeks after booster vaccination), at 1.57 ± 0.52 and 0.31 ± 0.17 for serotype Ia and III, respectively. There were a proportion of fish in the control group also displayed positive antibody measuring against *S. agalactiae* serotypes Ia and III at different time points.

### 3.4 Vaccine effectiveness in the farm setting

**Figure 6:**
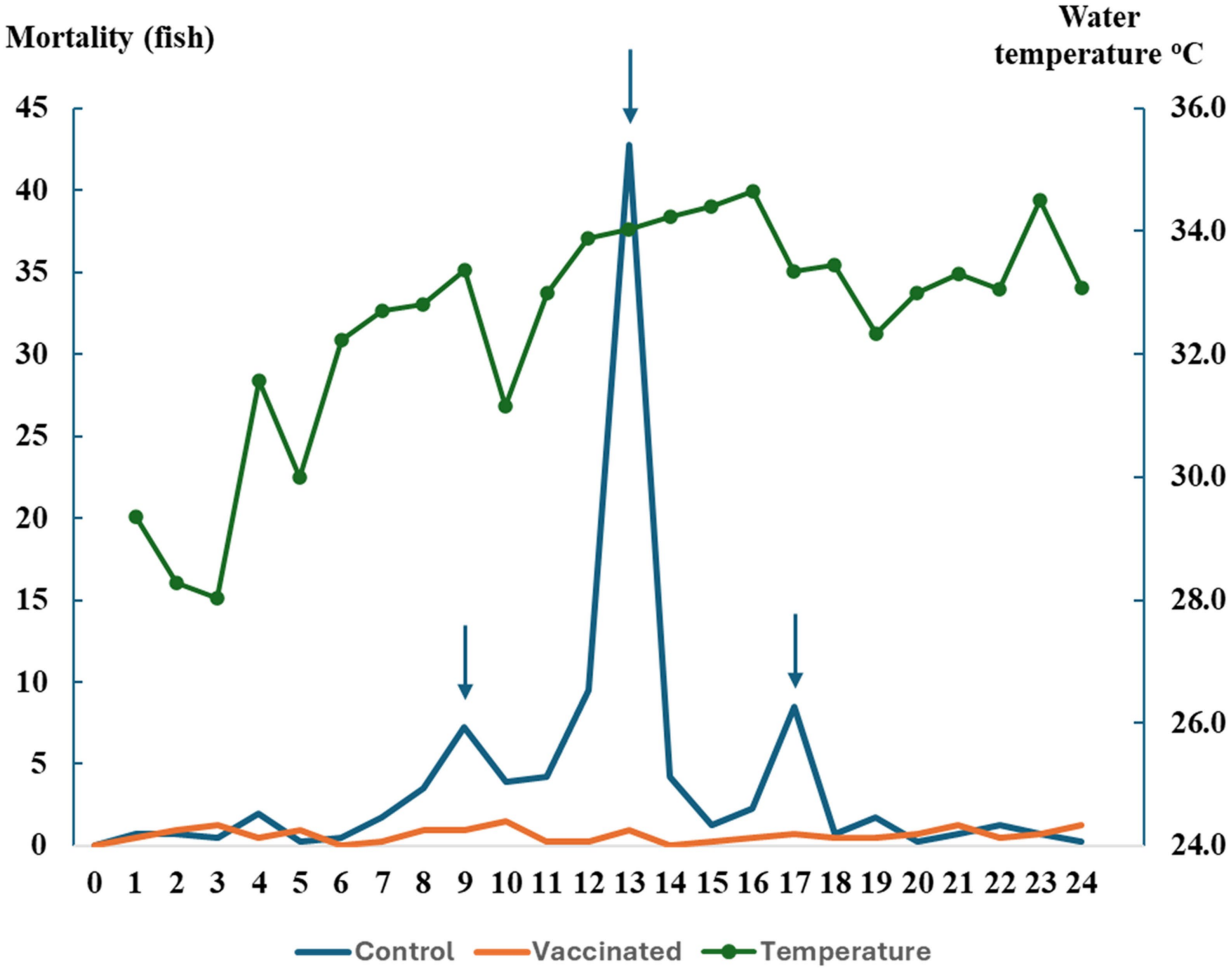
Average mortality of tilapia and water temperature (at 3:00 p.m.) by week of culture in the farm trial. Arrows indicate peak mortalities on week 9th, 13th, and 17th.

Figure 6 demonstrates the average weekly mortality of fish in the field trial while Table 2 summarizes survival rate of fish at the point of harvest. Overall, the vaccinated group experienced negligible mortality during the whole trial period. In contrast, the control group showed heavy mortalities at three noticeable time points at week 9, 13, and 17. The disease fish showed typical signs of streptococcosis including pop eyes and erratic swimming. The most severe mortality was recorded at week 13 at 42.8 ± 4.6 fish. At harvest, survival rates were recorded at 66.8 ± 6.1% and 94.5 ± 0.6% in the control and vaccinated group, respectively. These figures indicated significantly higher survival rate of the vaccinated group compared with the control group by chi-square test, *X^2^*(1, *N* = 2,400) = 294.49, *p* < 0.*0001.* The RPS in the vaccinated group was calculated as 83.4%.

**Table 2:** Survival rate (mean ± SD), harvest biomass and revenue of the crop at harvest according to group.

Group	Survival rate (%)	RPS (%)	Total biomass (kg)	Revenue (THB)
Control	66.8 ± 6.1	–	539.2	37,744
Vaccinated	94.5 ± 0.6****	83.4	783.4	54,838

Asterisk marks at the end of figures of the same growth parameter indicates significant difference between control and vaccinated group, ****p < 0.0001. Revenue was calculated based on the real average price of 70 THB/kg across sizes of fish.

### 3.5 Fish growth and production at harvest

Table 3 represents the monthly data of average biomass per cage (kg), average fish weight (g), average daily gain (g/day), and FCR over the culture period. Overall, there were no significant differences in average weight, and daily weight gain of fish between control and vaccinated group. However, recorded total biomass became divergent from week 16 of the trial, with the vaccinated group achieved an average harvest biomass at 195.9 ± 5.6 kg, significantly higher than the control group at 134.8 ± 15.2 kg. In contrast, FCRs calculated in the vaccinated group were significantly lower in the control group from week 16. At harvest, FCR in the control group was 2.00 ± 0.12, which was significantly higher that of the vaccinated group at 1.60 ± 0.03. In the end, the vaccinated group produced a total biomass of 783.4 kg, higher than the control group at 539.2 kg (Table 3).

**Table 3:** Growth performance (mean ± SD) of red tilapia during the field trial.

Week	Average biomass per cage (kg)		Average fish weight (g)	
	Control	Vaccinated	Control	Vaccinated
0	12.0 ± 0.1	12.0 ± 0.1	40.0 ± 0.2	39.8 ± 0.3
4	30.4 ± 2.8	32.4 ± 3.3	102.9 ± 9.2	111.7 ± 9.8
8	63.9 ± 6.1	63.1 ± 6.3	219.4 ± 18.1	219.2 ± 18.2
12	81.1 ± 2.4	87.7 ± 7.4	307.4 ± 6.8	307.7 ± 21.1
16	89.3 ± 12.0	123.8 ± 6.0**	425.8 ± 10.7	438.3 ± 8.0
20	110.6 ± 11.8	160.8 ± 4.7**	559.0 ± 16.5	573.2 ± 7.8
24	134.8 ± 15.2	195.9 ± 5.6**	691.6 ± 18.2	710.6 ± 12.6

Week	Average daily gain (g/day)		Cumulative FCR	
	Control	Vaccinated	Control	Vaccinated
0	–	–	–	–
4	2.25 ± 0.33	2.57 ± 0.35	1.46 ± 0.24	1.28 ± 0.17
8	4.16 ± 0.54	3.84 ± 0.53	1.33 ± 0.09	1.33 ± 0.13
12	3.14 ± 0.67	3.16 ± 0.38	1.67 ± 0.06	1.52 ± 0.12
16	4.23 ± 0.45	4.67 ± 0.57	2.06 ± 0.22	1.51 ± 0.06*
20	4.76 ± 0.23	4.82 ± 0.37	2.02 ± 0.14	1.53 ± 0.03*
24	4.74 ± 0.23	4.91 ± 0.29	2.00 ± 0.12	1.60 ± 0.03**

Asterisk marks at the end of figures of the same growth parameter indicates significant difference between control and vaccinated group. Significant levels *p* < * 0.05, ** 0.01.

### 3.6 Water quality in floating net cage units during the field trial

Table 4 summarizes water quality parameters in fish cages. From February to July, the temperature increased gradually (Figure 6) then became particularly high (over 32–33 °C) from week 8 of the trial, ranging from 27.9 to 34.9 °C. Overall, the period experienced high temperatures at an average of 32.5 °C. DO in the afternoon showed a range of 2.5 to 8.4 ppm and was 5.2 ppm on average. pH levels were consistent at 7–7.5 during the trial. Nitrogen measurement regarding TAN and NO_2_ demonstrated a rage of 0–0.5 ppm with averages of 0.2 ppm for each parameter.

**Table 4:** Water parameters inside the cage units during the field trial.

Parameter	Temperature (°C)	DO (ppm)	pH (ppm)	TAN (ppm)	NO_2_ (ppm)
Range	27.9–34.9	2.5–8.4	7–7.5	0–0.5	0–0.5
Average	32.5	5.2	7.2	0.2	0.2

### 3.7 Revenue and cost–benefit of the vaccine program

The cost of juveniles was 6 THB per fish. With 1,200 fish stocked in each treatment, the total seed cost per treatment group was 7,200 THB (Table 5). This corresponded to average costs for seed at 13.35 THB/kg of harvested fish in the control group and 9.19 THB/kg in the vaccinated group. The feed cost was 26.50 THB/kg. In the control group, a total of 974.6 kg feed was used, resulting in 25,826 THB (equivalent to 47.90 THB per kilogram fish produced). In parallel, fish in vaccinated group consumed 1,176.6 kg feed, costing 31,180 THB (39.80 THB/kg fish). On average, the HKV application reduced the production cost per kilogram of tilapia to 53.82 THB, compared to 61.24 THB in the control group.

**Table 5:** Summary of cost–benefit analysis.

Metric	THB/crop		THB/kg	
	Control	HKV	Control	HKV
Incremental costs				
Fry	7,200	7,200	13.35	9.19
Feed	25,826	31,180	47.9	39.8
Vaccine	0	3,784	0	4.83
Total	33,026	42,165	61.24	53.82
Incremental outcomes				
Revenue	37,744	54,838	70	70
Profit	4,718	12,673	8.75	16.18
Profit-Cost Ratio (PCR)	0.14	0.30		
Profit Margin (%)	12.5	23.11		

Only incremental costs, revenue, and profit were used for comparisons; identical costs across treatments, such as cage depreciation, electricity, and feeding labor, were excluded.

The vaccinated group generated a revenue of 54,838 THB, surpassing the control group’s by approximately 17,000 THB, or 45% (Table 5). The vaccination program increased an additional investment of 3,784 THB (or 4.83 THB/kg fish at harvest), accounting for nearly 9% of total cost. Although vaccination incurred higher cost, the vaccinated group returned a profit of 12,673THB, or 16.18 THB/kg fish at harvest. In contrast, the control group had a lower profit at 4,718 THB, equivalent to 8.75 THB/kg, although the initial investment was substantially lower by around 11,000 THB. In the HKV group, the PCR was 0.30, more than double that of the control group at 0.14. This translated into profit margins of 23.11 and 12.50% for the vaccinated and control groups, respectively.

## 4 Discussion

This study provides lab- and field-based evidence supporting autogenous vaccination as a viable alternative to antibiotics for controlling streptococcosis in tilapia. Despite the availability of commercial vaccines, their adoption in Asian aquaculture is alarmingly low, at around 1% (39). This can be attributed to inadequate vaccine distribution and skepticism among producers regarding their effectiveness and cost, especially for low-value fish species (52). Vaccine development to prevent *S. agalactiae* faces a substantial challenge due to its diversity in serotypes and strains, preventing commercial vaccines to fully cover the characteristics of local strains. For example, the farm involved in this study reported having previously tested a commercial vaccine against streptococcosis; However, it provided insufficient protection under their specific farming conditions. To address this issue, our research focused on developing a back-to-basics, a locally produced autogenous bivalent vaccine targeting *S. agalactiae* serotypes Ia and III, previously isolated from affected farms, and on demonstrating its efficacy and effectiveness while elucidating the long-term kinetics of the humoral antibody response under both laboratory and farm conditions.

Formalin-killed vaccines are the most extensively studied inactivated *S. agalactiae* vaccines for tilapia, with diverse application strategies across developmental stages and delivery methods (Table 6). In contrast, the use of HKV against *S. agalactiae* has been less explored, and their efficacy relative to FKV remains poorly documented. This study, for the first time, demonstrated that HKV and FKV against *S. agalactiae* elicited comparable specific antibody responses and protective efficacy, consistent with findings from studies on inactivated vaccines against tilapia lake virus (TiLV) (53). Previous studies have emphasized the importance of incorporating extracellular products from cultured bacteria to enhance the efficacy of FKV (28, 54). However, this often requires labor-intensive separation of killed cells from extracellular materials prior to formulation. In contrast, HKV offers a simpler alternative, as they can be produced without such separation, thereby retaining extracellular components within the bacterin. This approach streamlines production, reduces costs, and enhances accessibility, particularly in laboratories with limited infrastructure. The quantity and immunogenic potential of these extracellular components, however, warrant further investigation. Moreover, HKV may provide a practical and rapidly adaptable solution for addressing emerging local strains and serotypes, supporting their potential for broader application in aquaculture.

**Table 6:** Summary of studies for *S. agalactiae* killed vaccines in tilapia.

Kill method	No. of serotype(s)	Delivery	Adjuvants/ other materials	Booster	Fish size	Duration**	Efficacy	Ref.
Formalin (Lab)	1 (NS*)	Injection, Immersion	Extracellular products	No	~30 g	30–64 days	RPS Injection - 80% Immersion – 34%	(28)
Formalin (Lab)	1 (NS)	Injection	Extracellular products	No	45.5 ± 12.2 g	47, 90, 180 days	RPS 67, 62 and 49% - decreased overtime.	(71)
Formalin (Lab)	1 (NS)	Injection	No	Single booster	~20 g	30 days	RPS 96.6%	(72)
Formalin (Lab)	1 (NS)	Oral	Freund’s incomplete adjuvant (FIA)	Single booster	100 ± 10 g	28 days	Survival rate 100%	(73)
Formalin (Lab)	1 (NS)	Injection	Montanide ISA763AVG + phosphoglycerate kinase (PGK) / ornithine carbamoyl-transferase (OCT)	Single booster	38 ± 6 g	42 days	RPS Non-adjuvant 47.1% With PGK 82.4% With OCT 58.8%	(74)
Microwave (Lab)	1 (NS)	Injection, immersion	Extracellular products	No	5.6 ± 1.5	30 days	RPS Injection 56% Immersion 24%	(75)
Formalin (Lab)	1 (NS)	Injection	Extracellular materials	No	~250 g	14–21 days	Immunity transfer protected larvae that decreased overtime.	(54)
Hydrogen peroxide (Lab)	1 (Ib)	Injection	Aluminum hydroxide/ FIA	No	22.39 ± 1.71 g	28 days	RPS Non-adjuvant 40.7% Aluminum hydroxide 59.3% FIA 77.8%	(31, 32)
Formalin (Lab)	1 (NS)	Injection	No	Single booster	20 ± 3 g	28 days	RPS 92.3%	(76)
Heat (Lab)	1 (NS)	Immersion	Retaining extracellular products	No	15.62 ± 0.45	21 days	RPS 52.9–70.5%	(30)
Formalin (Lab)	1 (NS)	Immersion, Injection, Oral	Montanide ISA 763A VG Montanide ISA 1312 VG	No	9.85 ± 0.35 g	15 days	RPS Immersion 66.67% Oral 71.67% Injection 80%	(77)
Formalin (Lab)	1 (Ia)	Immersion	Mixture of peptidoglycan, palm oil, carbomer, glycerinum, diethanol amine in water	No	0.1, 0.5, 1.0 g	14, 30, 60, 90 days	Generally higher RPS in bigger fish that decreased overtime	(29)
Formalin (Lab/Field)	2 (Ia & III)	Injection	No	No	Lab −10.3 ± 3.7 g Field – 20 g	Lab – 60 days Field – 4-5 months	Lab RPS 35.4–57.1% against individual serotypes Field survival rates 77.4–97.1%	(44)
Formalin (Lab)	1 (NS)	Oral	Sodium alginate	No	25 ± 2 g	30 days	RPS 92%	(78)
Formalin (Lab)	1 (NS)	Injection	*β*-glucan / Alkoxy glycerol (AKG)	No	12 ± 2 g	28 days	RPS β-glucan+AKG 71.43% higher than separate adjuvant and non-adjuvant vaccines	(79)
Heat (Lab/Field)	2 (Ia & III)	Injection	Retaining extracellular products	Single booster	Lab – 26.3 ± 9.1 Field – 40 g	Lab – 56 days Field – 6 months	Lab RPS 86.8–100% against individual serotypes Field RPS 83.4%	This study

*NS, Serotype(s) not specified. **Duration of vaccine trial before challenge experiment or harvest point in field experiment.

The production of specific antibodies is essential to the immune response in fish, playing a crucial role in disease mitigation. In tilapia, the identified immunoglobulin classes include IgM, IgT, and IgD (55), with IgM being predominant for humoral immunity (56). In addition, the availability of commercial anti-tilapia IgM antibodies led to the selection of IgM levels for antibody response assessment. In our lab experiment, we observed a similar pattern of antibody response against serotype Ia and III in both HKV and FKV groups. The rise of specific IgM antibody levels in serum, that gradually declined toward week 4, indicates a short-lived specific antibody response in fish following the first injection dose. Aligning with many vaccine studies for tilapia, vaccinated tilapia in this research exhibited significant IgM levels in the first or second week of immunization (30, 57–59). The decline of IgM levels within a month suggests the need for a booster dose to enhance immune memory and to sustain specific antibody levels. In this study, administration of a booster dose on day 28 resulted in the rapid resurgence of high IgM levels against both serotypes of the serum. This evidence suggests the successful activation of the adaptive immune system and the maturation of memory immune cells specific for both serotypes of *S. agalactiae* following primary vaccination. The rapid and robust increase in specific antibody levels following the booster dose further supports the establishment of acquired immunological memory. Notably, vaccinated fish maintained significantly elevated IgM levels against serotype Ia and at least above threshold levels against serotype III prior to the challenge on day 56, indicating the potential of the vaccine to confer prolonged protection against streptococcosis.

Fish mucus containing mucosal antibodies serves as the first physical and immunological barrier to prevent invasion of infectious agents (60, 61). In our study, mucosal IgM rose in response to the stimulation of each vaccine injection, with similar pattern as observed in serum IgM. However, these mucosal IgM levels were relatively low and short-lived compared to those in the serum. The increase in mucosal IgM after injection vaccination were reported in previous studies (48, 53). This phenomenon may be attributed to the translocation of antibodies from the systemic to mucosal compartments or to the activity of antibody secreted B cells residing in mucosa-associated lymphoid tissues (MALTs) located in the skin and gills (48, 60). It is important to note that IgT was not evaluated in this study due to the limited availability of specific detection tools. Assessing mucosal IgM alone may only partially reflect the complexity of the fish’s immune system, highlighting the need for further investigation.

The immersion exposure of vaccinated fish with a sublethal dose of *S. agalactiae* aimed to replicate natural exposure conditions during the disease season by simulating infection through epithelial entry points such as the gills, skin, and gastrointestinal tract. Water-borne *S. agalactiae* can be ingested and subsequently adhere to the gut mucosal surface. Following colonization, the bacteria may breach the gastrointestinal epithelium and disseminate via the circulatory system (62). In contrast, infection via the skin may require prior damage to the epithelial barrier (50). Fish mucosa-associated immune tissues (e.g., gills, gut, and skin) help prevent further spread of invaders through both innate and adaptive immune responses (63). These mucosal tissues harbor both T and B cells and can mount effective antibody responses upon stimulation (60, 64). Studies have shown that immersion exposure of live *S. agalactiae* induced increased antibody levels in tilapia serum (25, 65).

Following exposure, both HKV and FKV groups displayed a notable rise in IgM after contact with *S. agalactiae*. This rapid response in vaccinated groups may indicate the efficient establishment of specific immune memory in tilapia after vaccination. The first-time exposure to *S. agalactiae* antigens in the control group also showed elevated humoral immune response of the fish to live pathogens, albeit at a lower degree. While immersion exposure incurred negligible mortality for efficacy interpretation, the distinct differences in systemic and mucosal immune responses between fish groups can reflect a higher possibility of vaccinated fish to overcome the infection. This finding aligns with a previous study on vaccination in Asian seabass (*Lates calcarifer*), which reported a correlation between elevated antibody levels and increased survival rates following experimental challenges (66).

The strong immune response elicited by the injection vaccines resulted in high survival rates during the injection challenge with a lethal dose of different *S. agalactiae* serotypes. This indicates the high protective capability of the bivalent vaccine against both serotypes of this pathogen. While survival rates were marginally higher with the formalin-killed vaccine (FKV) compared to the heat-killed vaccine (HKV), our current research found no significant difference in survival between the two, as determined by the log-rank test. Previous studies also attempted to compare HKV and FKV, but the superiority of one over the other might be related to pathogens, fish species, and vaccine administration methods. For example, FKV and HKV were comparable in protection against TiLV in tilapia (53), whereas the HKV showed higher efficacy against motile aeromonads in rainbow trout (*Oncorhynchus mykiss*) (67).

Because the vaccine was prepared by heat, some protective antigens may have been partially denatured during processing. Antigen integrity after heat treatment was not directly evaluated in this study and therefore warrants further investigation. Nevertheless, the comparatively high survival relative to the FKV in the laboratory trial is encouraging and suggests that key protective epitopes were sufficiently retained to confer protection. Given its high protective capacity, coupled with simpler and more affordable production, HKV was therefore selected for further assessment.

In the field, after being administered with HKV, red tilapia demonstrated high levels of serum IgM antibody response against both serotypes, sharing a similar pattern as observed in the laboratory trial. Interestingly, a sub-set of fish in the control group demonstrated slightly positive *S. agalactiae*-specific IgM (at the *0.05* significance cut-off) against both serotypes from the third week of the trial. In addition, some fish displayed exophthalmia, skin hemorrhages in the head region and at the fin base, as well as ulcers beneath the jaw. These signs suggest an ongoing *S. agalactiae* infection during the trial period, although the responsible serotype(s) could not be identified due to limited facilities at the farm. Moreover, high and fluctuating temperatures can increase tilapia’s susceptibility to infections caused by *S. agalactiae*, as these conditions promote bacterial growth while potentially inducing stress to the fish (17, 18). In our study, peaks of mortality in the control group were found to coincide with temperatures exceeding 33 °C, thereby supporting the probability of an ongoing infection.

A previous study demonstrated desirable survival rates (77.4 to 97.1%) against the disease in farm settings via injection FKV *S. agalactiae* serotype Ia and III, with notable differences in survival rates observed during high-temperature period from March to August (44). Our finding also reflected a significant divergence in survival rates during the hot season, with the vaccinated group showing high protective capabilities at 94.5%, compared to that of 66.8% in control. While confirming the prevalence of *S. agalactiae* through diagnosis is important, the combination of sero-surveillance data, observed clinical signs, environmental factors, and survival rates collectively showcases the effectiveness of the vaccination program.

The immune response of tilapia against serotype III appeared less immunogenic compared to serotype Ia, despite vaccine formulation contained similar antigen concentration. This was evident from the faster decline in OD measurements for serotype III following the injections in the lab, and more notably in serum IgM levels found in the field trial. In addition, mucosal IgM levels against serotype III were much shorter-lived, whereas the response against serotype Ia was more consistent. A likely reason for serotype III being less immunogenic and inducing a shorter duration of immunity could be related to differences in the structure or composition of its capsular polysaccharides (CPS), which affect antigen recognition and immune activation in the host (68). In clinical trials of multivalent CPS *S. agalactiae* vaccines, serotype III was consistently observed to be less immunogenic than other serotypes (69, 70). Furthermore, a previous bivalent *S. agalactiae* vaccine (serotype Ia and III) also revealed lower optical readings in ELISA for those against serotype III (44). Given the complexity of the adaptive immune system in fish, our study - which primarily focuses on antibody responses as a measure of humoral immunity, may not fully capture the variations in immune responses elicited by different serotypes. Therefore, to gain a more comprehensive understanding, it is essential to evaluate both cell-mediated and humoral immunity.

In the farm trial, the high survival rate of vaccinated fish improved revenue by approximately 45%. The average weight of fish and average daily gain were similar between control and vaccine treatments. However, FCR in the control group was significantly higher than that of the vaccinated group. These outcomes indicated that the improved yield was as a result of improved survival, with minimal effect on individual growth.

In this study, a CBA was undertaken. The production costs for the vaccine in our study were relatively low (primarily comprising expenditures related to materials, labor, energy, storage, and quality control). On contrary, the cost of equipment and labor for injection of individual fish was significantly higher. The estimated cost for small-scale vaccine production in this study may not fully reflect an industrial setting. In addition, regulatory costs remained difficult to quantify. To account for uncertainty in the costs, we adopted a conservative approach to pricing by keeping a high profit margin at 50% for the final vaccine price. Regardless, given the simplicity in production of HKV, the vaccine price can remain competitive in industrial scale.

The vaccination program has the potential to offset disease-related losses, which often represent a substantial challenge in tilapia farming. In the vaccinated group, the higher survival rate led to more efficient resource utilization, substantially reducing the average cost to produce each kilogram of fish compared to the control group. As a result, the vaccinated group was more profitable, with a PCR about two times higher than that of the control group, despite the additional costs of vaccination.

The small scale of this trial (1,200 fish per group) may inflate production costs compared with economy-scale production. As indicated by personal communication with the farm owner, the production cost in this small-scale trial was higher than would be expected in large-scale, real-world production (i.e., 30–50 cages; cage size 5 × 5 × 2.5 m). This may be the primary reason the profit was not optimal, therefore rendering CBA less robust. Nonetheless, the profitability advantage in the vaccinated group demonstrated the attractive cost-effectiveness of the vaccine program. This evidence may encourage farmers to adopt fish vaccine in tilapia aquaculture to mitigate streptococcosis. Future work should aim for a more sensitive analysis that incorporates vaccine and labor costs, various farming scales, mortality rates, and market prices to provide a more precise CBA.

The high survival rate of the vaccinated group in the field trial is promising, probably thanks to target-tailored vaccine that protected the fish from local serotypes. Due to geographical limitations, the autogenous bivalent vaccine may need reformulation to match with local strains of *S. agalactiae* in different areas. Additionally, the specific environmental conditions by different locations and farm practices such as water quality, temperature, and the presence of other pathogens can significantly affect disease outbreaks and fish immune responses. Therefore, future research should assess the vaccine’s performance in diverse geographical settings to strengthen its usefulness.

Injection vaccination is still considered labor-intensive and costly for tilapia aquaculture, which may hinder the widespread adoption by producers. Nonetheless, the HKV has the potential for lower production costs than FKV, making it a promising first step toward more affordable vaccines. To enhance feasibility, injection vaccination could be used to target vulnerable groups, particularly during seasonal outbreaks, on farms with a history of severe disease, or in high-value stocks such as broodstock. This approach would require a good understanding of disease patterns and careful record-keeping to ensure timely vaccine application. In low- and middle-income countries, autogenous vaccines should be considered as part of an informed veterinary health management program to improve rapid accessibility and uptake. Finally, this bivalent vaccine could be reformulated and evaluated for alternative delivery routes, such as immersion or oral administration, which are generally more practical and attractive to farmers.

## 5 Conclusion

The locally formulated bivalent HKV targeting *S. agalactiae* serotype Ia and III stimulated specific immunity in tilapia and conferred high survival rates under both controlled laboratory and field conditions. In the field application, the vaccine increased revenue by approximately 45%, and more than doubled the profit–cost ratio compared to the non-vaccinated group. The vaccine offers a promising non-antibiotic and feasible approach to mitigate *S. agalactiae* in tilapia aquaculture. Therefore, we recommend that killed vaccines, including autogenous formulations, particularly HKV, be locally authorized and actively promoted as a practical, context-appropriate alternative to antibiotics in aquaculture. This is particularly important in low- and middle-income countries where antibiotic overuse is common and access to vaccines remains restricted by regulatory and operational challenges.

## Data Availability

The original contributions presented in the study are included in the article/supplementary material, further inquiries can be directed to the corresponding author/s.

## Footnotes

1 PHARMAQ, https://www.pharmaq.com/en/pharmaq/our-products/

2 MSD Health, https://www.msd-animal-health-me.com/species/aqua/products/

3
https://corporate.virbac.com/

## Appendix

**Table A1:** Cost for vaccine production.

Cost	Cost per unit	#Unit	Unit	Total cost (THB)	Notes
Medium	90.00	1	Liter	90.00	
Electricity for autoclave	4.00	1.5	KW	6.00	3KW machine, 0.5 h operation
Electricity for water baht	4.00	0.45	KW	1.80	Estimated for 1.5 h operation
Electricity for shaker	4.00	6	KW	24.00	6KW/day machine, 24 h operation
Labor	600.00	1	day	600.00	
Other materials	40.00	1		40.00	Falcons, agar plates, and related miscellaneous materials
Total cost per liter				761.8	
Selling price at 50% profit margin				1,523.6	
Total cost per dose				0.30	Total 5,000 dose per 1 L, 0.2 ml per dose

**Table A2:** Cost for injection vaccination.

Cost	Cost per unit	#Unit	Unit	Total cost (THB)	Cost proportion (%)	Notes
Labor cost	400.00	5	People	2,000.00	53	2 doses = 2 half days (Cost per unit = per day)
Vaccine gun and equipment	290.00	2	Times	580.00	15	
Other equipment	100.00	2	Times	200.00	5	
Equipment Maintenance	100.00	2	Times	200.00	5	
Vaccine	0.30	2,640	Doses	804.46	21	2 doses * 1200 fish * 1.1 extra volumes
Total cost				3,784.46	100%	
